# A Text-Book upon the Pathogenic Bacteria, for Students of Medicine and Physicians

**Published:** 1896-07

**Authors:** 


A TEXT-BOOK UPON THE PATHOGENIC BACTERIA, for Students of Medicine and Physicians. By JOSEPH MCFARLAND, M. D., Demonstrator of Pathological Histology and Lecturer on Bacteriology in the Medical Department of the University of Pennsylvania; Pathologist to the Rush Hospital for Consumption and Allied Diseases. Octavo, pp. 359. With 113 illustrations. Price, $2.50 net. Philadelphia: W. B. Saunders, 925 Walnut street. 1896.

A new text-book on bacteriology in these days of prolific publication needs some distinguishing features to make it a success and acceptable to the student world and profession at large. The author of this work has departed from the beaten paths and has produced a work of great benefit and convenience to the busy practitioner, serving at the same time as a text-book invaluable to the laboratory students.

The pathogenic bacteria only are discussed by the author. The first part of the book conveys to the reader a concise account of the technical procedures necessary in the study of bacteriology and a brief description of the life history of the important pathogenic bacteria.

Part two is devoted to the different infectious diseases resulting from well-known bacteria, with the origin of symptoms and cause of death. It seems very desirable to have a work where one can turn to any infectious disease, as tuberculosis, for instance, and study the etiology and pathology from the bacterial point of view, the bacteria being secondary to the disease. In the same way are discussed chronic inflammatory diseases, glanders, leprosy, syphilis, actinomycosis, mycetoma, farcin du boeuf and rhinoscleroma. Under the toxic diseases are discussed diphtheria, tetanus, rabies, anthrax, typhoid fever, cholera and pneumonia, while under the septic diseases are discussed relapsing fever, influenza, malignant edema, measles, bubonic plague, tetragenus, chicken cholera, mouse-septicema, anthrax and typhus murium. The descriptions are all well rendered, clear and concise, the illustrations carefully selected and the result is a most commendable little volume.

The typographical work is admirably executed. W. C. K.

